# Pathognomonic oral profile of Enamel Renal Syndrome (ERS) caused by recessive *FAM20A* mutations

**DOI:** 10.1186/1750-1172-9-84

**Published:** 2014-06-14

**Authors:** Muriel de la Dure-Molla, Mickael Quentric, Paulo Marcio Yamaguti, Ana-Carolina Acevedo, Alan J Mighell, Miikka Vikkula, Mathilde Huckert, Ariane Berdal, Agnes Bloch-Zupan

**Affiliations:** 1 Laboratory of Molecular Oral Pathophysiology, INSERM UMRS 1138, Cordeliers Research Center, Paris, France; 2 Center of Rare Malformations of the Face and Oral Cavity (MAFACE), Hôpital Rothschild, Paris, France; 3 Laboratory of Human Molecular Genetics, de Duve Institute, Université catholique de Louvain, Brussels, Belgium; 4 Oral Care Center for Inherited Disease, University Hospital of Brasilia, University of Brasilia, Brasilia, Brazil; 5 Department of Dentistry, Health Sciences School, University of Brasilia, Brasilia, Brazil; 6 School of Dentistry, The University of Leeds, Leeds, UK; 7 Faculty of Dentistry, University of Strasbourg (UdS), Strasbourg, France; 8 Reference Centre for Orodental Manifestations of Rare Diseases, Pôle de Médecine et Chirurgie Bucco-Dentaires, Hôpitaux Universitaires de Strasbourg (HUS), Strasbourg, France; 9 Fédération de Médecine Translationnelle de Strasbourg (FMTS), Université de Strasbourg, Strasbourg, France; 10 Institute of Genetics and Molecular and Cellular Biology (IGBMC), CNRS UMR 7104 - Inserm U 964, Illkirch, France; 11 Laboratoire de Génétique Médicale, UMRS 1112, Faculty of Medicine, UdS, Strasbourg, France

**Keywords:** Enamel Renal Syndrome (ERS), Amelogenesis Imperfecta and Gingival Fibromatosis syndrome (AIFGS), Enamel Dysplasia with Hamartomatous atypical Follicular Hyperplasia Syndrome (EDHFH), Amelogenesis Imperfecta, Enamel defect, Delayed tooth eruption, Intra-pulpal calcification, Gingival hyperplasia, FAM20A

## Abstract

Amelogenesis imperfecta (AI) is a genetically and clinically heterogeneous group of inherited dental enamel defects. Commonly described as an isolated trait, it may be observed concomitantly with other orodental and/or systemic features such as nephrocalcinosis in Enamel Renal Syndrome (ERS, MIM#204690), or gingival hyperplasia in Amelogenesis Imperfecta and Gingival Fibromatosis Syndrome (AIGFS, MIM#614253). Patients affected by ERS/AIGFS present a distinctive orodental phenotype consisting of generalized hypoplastic AI affecting both the primary and permanent dentition, delayed tooth eruption, pulp stones, hyperplastic dental follicles, and gingival hyperplasia with variable severity and calcified nodules. Renal exam reveals a nephrocalcinosis which is asymptomatic in children affected by ERS. *FAM20A* recessive mutations are responsible for both syndromes. We suggest that AIGFS and ERS are in fact descriptions of the same syndrome, but that the kidney phenotype has not always been investigated fully in AIGFS. The aim of this review is to highlight the distinctive and specific orodental features of patients with recessive mutations in *FAM20A*. We propose ERS to be the preferred term for all the phenotypes arising from recessive *FAM20A* mutations. A differential diagnosis has to be made with other forms of AI, isolated or syndromic, where only a subset of the clinical signs may be shared. When ERS is suspected, the patient should be assessed by a dentist, nephrologist and clinical geneticist. Confirmed cases require long-term follow-up. Management of the orodental aspects can be extremely challenging and requires the input of multi-disciplinary specialized dental team, especially when there are multiple unerupted teeth.

## Classification

Amelogenesis imperfecta (AI) is a genetically and clinically heterogeneous group of inherited dental enamel defects. The enamel defects can be quantitative and/or qualitative. Reduced enamel thickness, typically with normal hardness is classified as hypoplastic AI, whereas reduced hardness, discoloration with normal thickness is termed hypomineralised AI, that incorporates hypocalcified and hypomaturation AI subtypes. Fourteen different AI subtypes have been described, distinguished by clinical description and mode of inheritance, although increasingly the emphasis is on genetic classification as insight into the underlying molecular changes increases. All inheritance patterns have been reported, including dominant, recessive and X-linked inheritance with various degrees of penetrance [1,2]. AI, commonly described as an isolated trait, may be observed concomitantly with a number of variable dental and/or systemic disorders [3,4]. Initially described by MacGibbon in 1972 [5], many cases with enamel defects associated with nephrocalcinosis have since been reported, either under the name “Amelogenesis imperfecta and nephrocalcinosis” [5-15], or alternatively, “Enamel Renal Syndrome” (ERS, MIM#204690) [16].

Forty years after MacGibbon’s case report, next generation sequencing was used to identify recessive mutations in the *FAM20A* (family with sequence similarities 20 member A) gene in 17 families with ERS [17,18]. *FAM20A* mutations were also identified as the cause of “Amelogenesis Imperfecta and Gingival Fibromatosis Syndrome” (AIGFS, MIM#614253) [19-23]. A detailed review of the clinical aspects of patients affected by these two syndromes reveals that the dental phenotype in both cases is in fact the same and that the kidney phenotype has not been investigated fully in AIGFS patients. Therefore recessive *FAM20A* mutations lead to only one disease with distinctive oral phenotype frequently associated with renal calcification.

## Epidemiology

Collectively, the various forms of AI are common, with prevalence rates varying from 1/700 to 1/14,000 in newborns, depending on the population studied [24,25]. A review of the clinical cases in the literature indicates that AI associated with the key features of ERS/AIGFS has been reported but named differently: only AI [26,27], AI with inter-radicular dentine dysplasia [28], AI with gingival fibromatosis [20,22,29,30], AI with odontogenic fibroma-like hamartomas around non-erupted teeth [31-35], AI with crown resorption [36] and AI with unerupted teeth [37]. To date, 34 articles have reported ERS-like cases (Table 1 and Additional file 1). Only 16 included a complete analysis of both dental/oral and renal phenotypes. Since the renal status was not systematically analyzed in these cases, ERS has been underestimated. Nevertheless, all together less than one hundred cases have been reported in the world. The prevalence of ERS is still unknown. In Israel, where the prevalence of AI is 1/8000, a study conducted on 70.359 affected children reported only 1 autosomal recessive hypoplastic AI patient with eruption defects, crown resorption and pulpal calcification reminiscent of ERS [26].

**Table 1:** All reported cases with clinical features potentially describing Enamel Renal Syndrome

	**Number of reported**		**Oral phenotype**	**Kidney phenotype**	**Diagnosis reported**	***FAM20A *** **mutation**
	**Families**	**Cases**	**ERS pathognomic oral findings**	**Bilateral nephrocalcinosis**		
MacGibbon [5]	1	1	+	+	Generalized **enamel hypoplasia** and **renal** dysfunction.	expected
Chosack *et al.*[26]	2	4	+ (1/2)	Not investigated	**Amelogenesis imperfecta** among Israeli Jews and the description of a new type of local **hypoplastic autosomal recessive amelogenesis imperfecta.**	expected for family 2 only
Lubinsky *et al.*[10]	1	2	+	+	Syndrome of **amelogenesis imperfecta**, **nephrocalcinosis**, impaired renal concentration, and possible abnormality of calcium metabolism.	expected
Nakata *et al.*[28]	1	2	+	Not investigated	**Interradicular dentin dysplasia** associated with **amelogenesis imperfecta.**	expected
Ooya *et al.*[27]	1	1	+	Not investigated	Autosomal recessive rough hypoplastic **amelogenesis imperfecta**. A case report with clinical, light microscopic, radiographic, and electron microscopic observations.	expected
Van Heerden *et al.*[34]	2	2	+	Not investigated	**Amelogenesis imperfecta**: multiple impactions associated with odontogenic **fibromas (WHO) type**.	expected
Peters *et al.*[32]	1	1	+	Not investigated	Rough hypoplastic **amelogenesis imperfecta** with **follicular hyperplasia**.	expected
Hall *et al.*[9]	1	2	+	+	**Amelogenesis imperfecta** and **nephrocalcinosis** syndrome. Case studies of clinical features and ultrastructure of tooth enamel in two siblings.	expected
Phakey *et al.*[14]					Ultrastructural study of tooth enamel with **amelogenesis imperfecta** in AI**nephrocalcinosis** syndrome.	
Dellow *et al.*[7]	1	2	+	+	**Amelogenesis imperfecta**, **nephrocalcinosis**, and hypocalciuria syndrome in two siblings from a large family with consanguineous parents.	confirmed^3^
Raubenheimer and Noffke [38]	1	1	-	Not investigated	**Enamel dysplasia, hamartomas.**	not expected
Normand de La Tranchade *et al.*[12]	1	1	+	+	**Amelogenesis imperfecta** and **nephrocalcinosis**: a new case of this rare syndrome.	expected
Paula *et al.*[13]	1	1	+	+	Case report of a rare syndrome associating **amelogenesis imperfecta** and **nephrocalcinosis** in a consanguineous family.	confirmed^3^
Cetrullo *et al.*[6]	1	2	-	+	Two cases of familial hypomagnesemia with hypercalciuria and **nephrocalcinosis**: dental findings.	not expected
Feller *et al.*[35]	1	1	+	Not investigated	**Enamel dysplasia** with **odontogenic fibroma-like hamartomas**: review of the literature and report of a case.	expected
Fu *et al.*[39]	1	1	-	+	**Enamel-renal syndrome** associated with hypokalaemic metabolic alkalosis and impaired renal concentration: a novel syndrome?	not expected
Elisabeth *et al.*[40]	2	2	+ (1/2)	+	**Amelogenesis imperfecta** with **renal disease**--a report of two cases.	expected for case 1 only
Feller *et al.*[31]	1	1	+	Not investigated	**Enamel dysplasia hamartomatous atypical follicular hyperplasia**: review of the literature and report of a case.	expected
Roquebert *et al.*[33]	1	1	+	Not investigated	**Amelogenesis imperfecta**, **rough hypoplastic type**, **dental follicular hamartomas and gingival hyperplasia**: report of a case from Central America and review of the literature.	expected
Martelli-junior *et al.*[30]	1	4	+	No history of renal pathology (but not investigated)	Case reports of a new syndrome associating **gingival fibromatosis** and **dental abnormalities** in a consanguineous family.	expected
Martelli-Junior *et al.*[11]	1	1	+	+	**Amelogenesis imperfecta** and **nephrocalcinosis** syndrome: a case report and review of the literature.	expected
Dos Santos *et al.*[29]	1^1^	4	+	Not investigated	Imaging evalution of the **gingival fibromatosis** and **dental abnormalities** syndrome.	confirmed^4^
Cho *et al.*[11]	4	4	+	Not investigated	Novel **FAM20A** mutations in hypoplastic **amelogenesis imperfecta**.	confirmed
Miloglu *et al.*[36]	1	2	+	Not investigated	Generalized familial crown resorptions in unerupted teeth.	expected
Kala Vani *et al.*[16]	1	1	+	+	**Enamel renal syndrome**: a rare case report.	expected
Hegde *et al.*[37]	1	2	+	Not investigated	Multiple Unerupted Teeth with Amelogenesis Imperfecta in Siblings.	expected
O’Sullivan *et al.*[20]	1^1^	4	+	Not investigated	Whole-Exome sequencing identifies **FAM20A** mutations as a cause of **amelogenesis imperfecta** and **gingival hyperplasia** syndrome.	confirmed
Jaureguiberry *et al.*[17]	16^2^	25	+	+	**Nephrocalcinosis** (**Enamel Renal Syndrome**) caused by autosomal recessive **FAM20A**	confirmed
Wang *et al.*[18]	3	5	+	+(1/3) Family 2	**FAM20A** Mutations Can Cause **Enamel-Renal Syndrome (ERS)**	confirmed
Cabral *et al.*[22]	1	12	+	-	Autosomal recessive **gingival hyperplasia** and **dental anomalies** caused by a 29-base pair duplication in the **FAM20A** gene	confirmed
Wang *et al.*[23]	2	3	+	+ (1/2) Neg. US in proband of Family 1	**FAM20A** Mutations Associated with **Enamel Renal Syndrome**	confirmed
Kantaputra *et al.*[21]	2	2	+	+	**Enamel-Renal-Gingival Syndrome** and **FAM20A** Mutations	confirmed

COF: Central Odontogenic Fibroma.

Neg. US: Negative ultrasounds analysis.

(+)/(−): cases positive vs. negative for the clinical features indicated. No sign: clinical features not mentioned or not investigated; + (1/2): positive in one case out of two; COF: Central Odontogenic Fibroma; ^1^Family previously described in Martelli-Junior *et al.*[32]; ^2^Two families previously described in Dellow *et al.*; Paula *et al*) [7,13]; ^3^Family sequenced in Jaureguiberry *et al.*[17]; ^4^Family sequenced in O’Sullivan *et al.*[22].

(Diagnosis element were highlight by boldface).

## Clinical description

**Figure 1:**
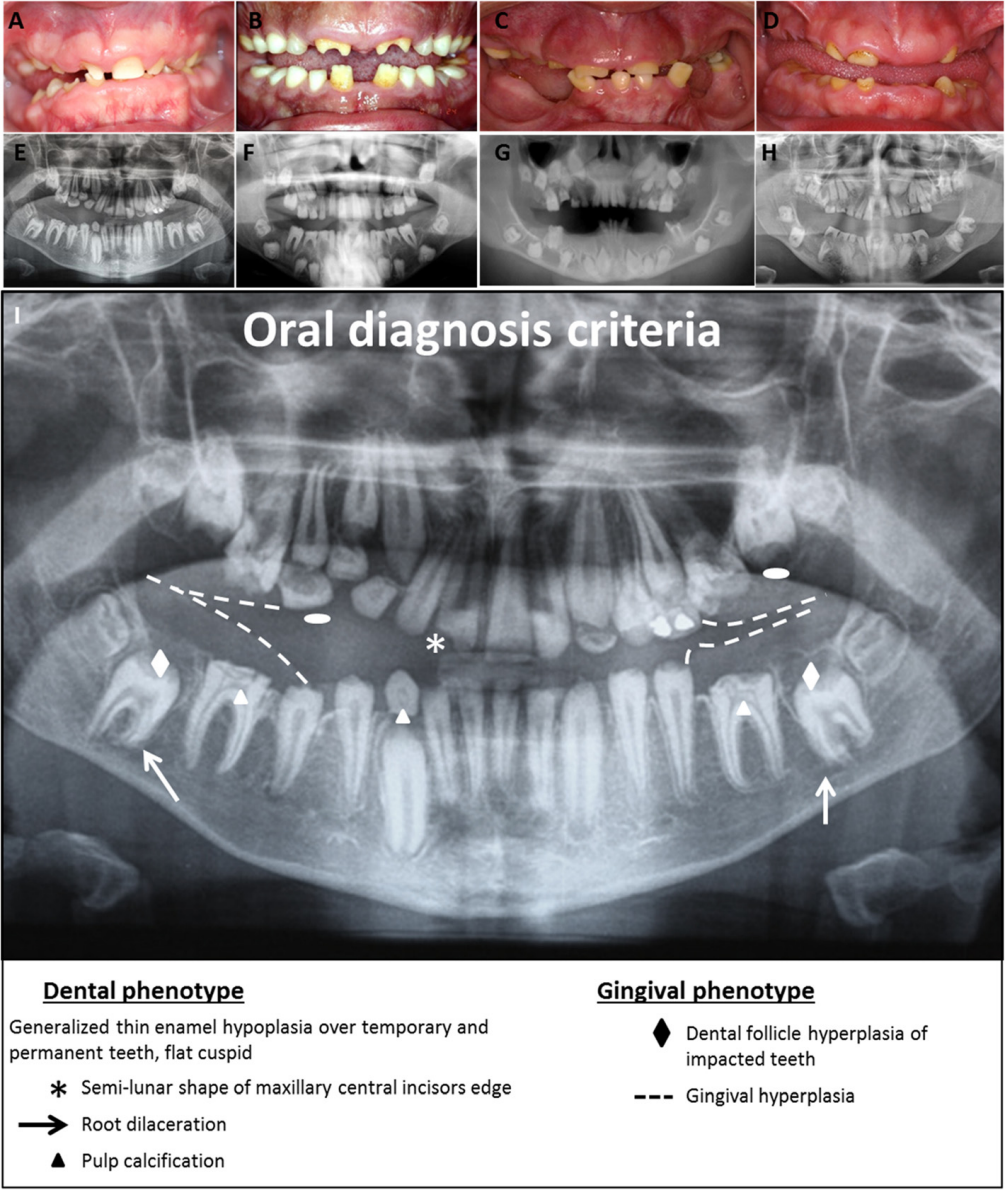
**Pathognomonic oral phenotypes in patients with Enamel Renal Syndrome. (A, B, C, D, E, F, G, H)** Oral views and corresponding orthopantomogram of 4 unrelated ERS patients, all with recessive mutations in *FAM20A*. Oral view showing hypoplastic AI, delayed tooth eruption, widely spaced teeth, flat cusps of posterior teeth, upper permanent central incisor crown resorption and gingival hyperplasia. Orthopantomograms show generalized absence of enamel, altered tooth eruption pathway, flat cusps of posterior teeth (white elipse), intra-pulpal calcification (white triangle), hyperplastic dental follicles (white diamond), and gingival hyperplasia (dashed line). Semi-lunar shape of the permanent right upper central incisor, crown resorption and root dilacerations (white arrow) are also present. Note the consistent oral features in all patients. **(I)** Summary picture of pathognomonic oral diagnosis criteria listed in orthopantomogram.

Patients are often referred to the dental practitioner due to enamel defects of the primary and permanent teeth. Pregnancy and delivery are uneventful, and no health problems are observed during the perinatal period and childhood. Patients with ERS/AIGFS present with a distinctive oral and renal phenotype (Figure 1).

### Oral phenotype

#### Dental phenotype

**Figure 2:**
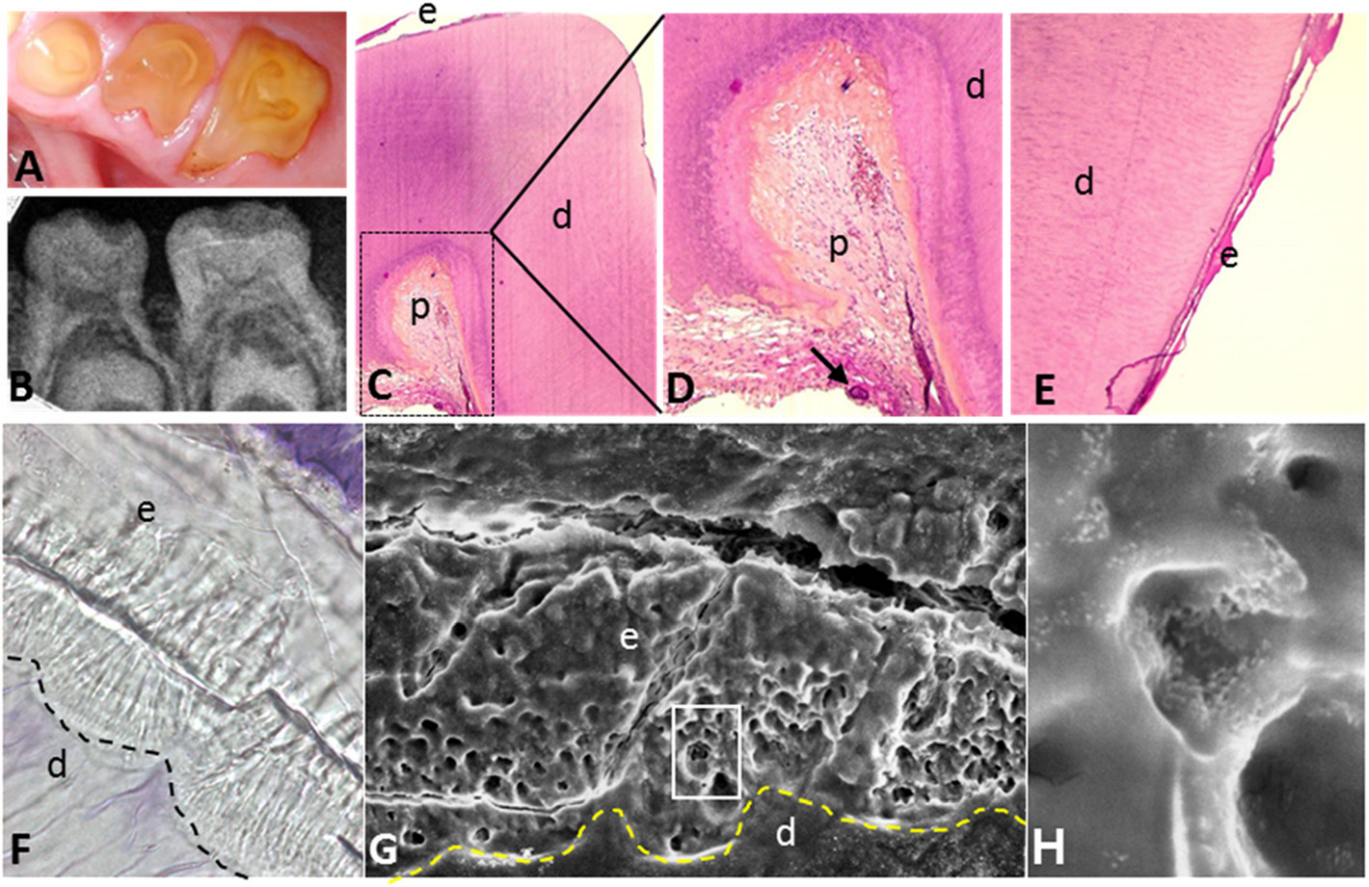
**Tooth phenotype of ERS patients. (A, B)** Oral and radiograph view show intra pulpal calcification and absence of enamel **(C)** H&E staining of a sagittal section from an extracted deciduous molar. **(D)** Magnified boxed area from C showing intra-pulpal calcification (black arrow). **(E)** Magnified view of the surface of the tooth presented in C showing a sparse thin hypoplastic enamel layer. **(F)** Sagittal section of a deciduous molar observed via optical microscopy (zoom ×840). Note normal tubular dentine, columnar arrangements close to the dentine indicating enamel rods contrasting with a generally disorganized enamel surface. Note the abnormal dentino-enamel junction which is less scalloped than normal (dashed line). **(G)** Magnified view of the area in F with scanning electron microscopy (zoom ×900). Note absence of normal enamel rod architecture and increased porosity in the enamel. **(H)** Magnified view of box in F (zoom ×10,000). e: enamel, d: dentin, p: dental pulp, DEJ: Dentin-enamel junction (dashed line).

**Figure 3:**
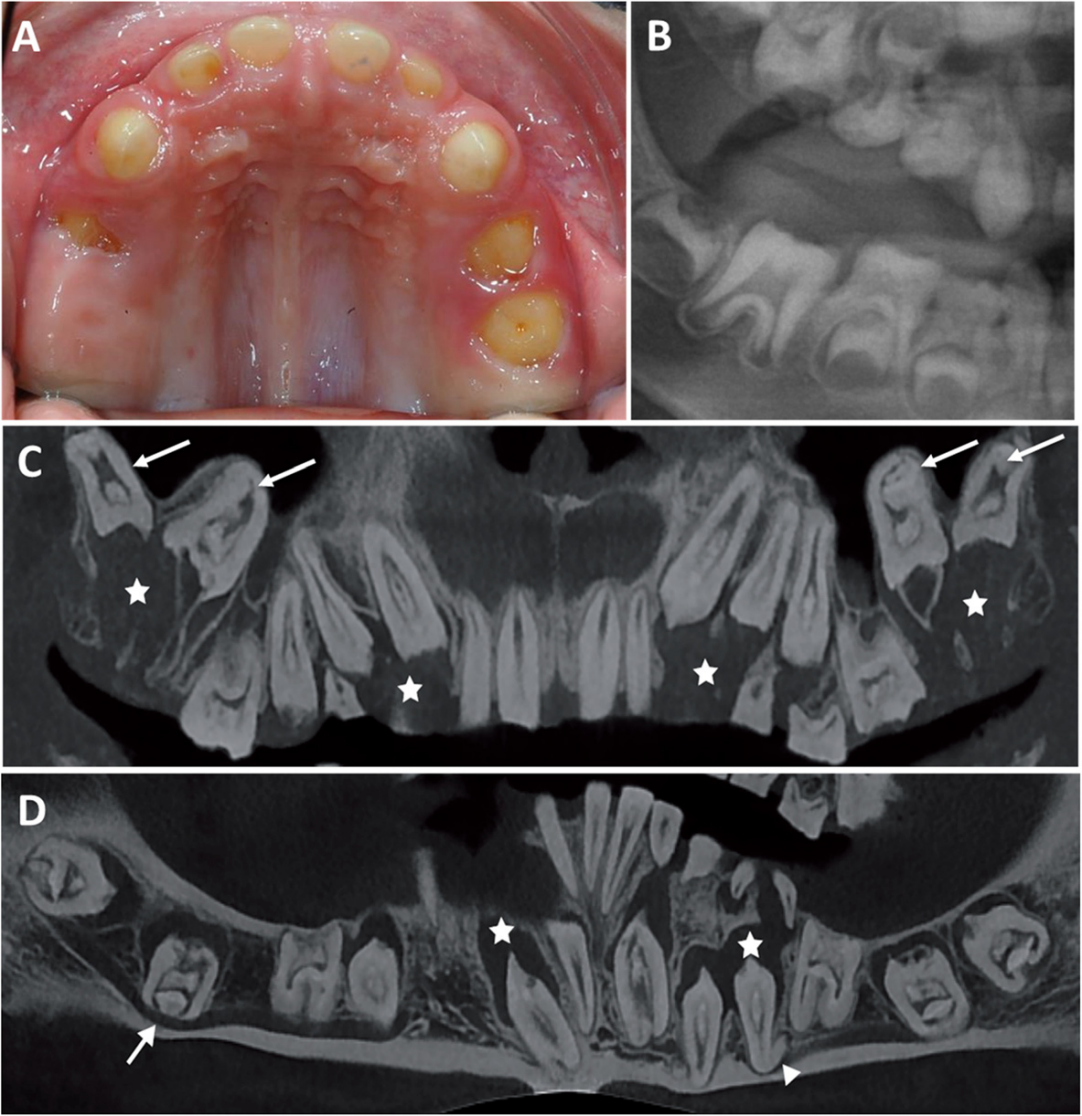
**Eruption anomalies of ERS patients. (A, B)** Oral and radiographic view of no erupted or partial erupted temporary molars in a 5 years old patient. **(C, D)** Cone beam radiograph of maxillary and mandible arch showing hyperplasia of dental follicle (star), dilacered root (triangle), inverted erupting pathway : maxillary molar is seen in the sinus and mandibular molar crossed cortical bone of mandible (arrow).

Oral and radiographic examination of patients shows consistent dental findings (Figure 1A-D). Teeth have a yellow-brown discoloration appearance and the surface can be either rough or smooth. Teeth are widely spaced suggesting microdontia, albeit with normal enamel hardness. Enamel layer thickness is drastically reduced, even sometimes absent in both primary and permanent teeth (Figure 2E). According to Witkop, such enamel dysplasia is classified as hypoplastic enamel type IF “rough hypoplastic” or IG “thin/agenesis enamel” [2]. Post-eruption wear of the irregular enamel matter may explain the extremely thin and smooth enamel that is frequently observed [9]. Dental pain or sensitivity on eating or drinking is not a prominent feature by contrast with other forms of AI. Molar crowns show flat occlusal surfaces without any characteristic cusp morphology. Semi-lunar shape of the upper central incisor edge is frequently, although not systematically, observed (Figure 1A-B). Eruption of permanent teeth is often delayed and sometimes completely impaired. Delayed eruption also occurs in primary teeth but less frequently (Figure 3A-B). These features suggest that early morphogenetic events during odontogenesis and eruption are affected in addition to amelogenesis.

Clinical examination must be completed with radiographic analysis (Figure 1E-I). Multiple calcification nodules (pulp “stones”) are discernible in pulp chambers in primary and permanent teeth, typically needle-shaped in the incisors and round in the posterior teeth (Figure 2A-B). The differential radiodensity expected between dentine and enamel is reduced or absent, indicating low mineral content in enamel or absence of enamel, respectively. Around non-erupted teeth, significant pericoronal radiolucencies, delineated by sclerotic margins, are observed. Despite the absence of eruption, root formation proceeds, leading to radicular dilacerations and shorter, sometimes curved, roots (Figure 3C-D). In addition, an abnormal eruption pathway is noted and is extremely clear for the second permanent molar with an inverted direction toward the mandibular canal or maxillary sinus. In some cases, thickening of the maxillary or nasal sinus is observed [29]. Hyperplasia of dental follicles appears to be associated with the abnormality of the eruption pathway and the absence of eruption (Figure 1I, Figure 3C). On some teeth, there is extensive localized root and/or crown resorption with partial replacement of the resorbed dentin by lamellar haversian bone or, in some places, globular structures comprised of incompletely coalesced concentric calcifications (Figure 1I) [12,18,34]. All teeth may be affected; however, posterior teeth are more frequently involved.

Light and scanning electron microscopy definitively established the severe decrease in thickness or absence of enamel (Figure 2C-H) [13]. Enamel appears as an irregular globular calcified layer with few or absent prismatic structures. Small and sparsely packed crystals are observed parallel to the surface, explaining porosity of the enamel [9,27,30]. An abnormal thick layer of what appears to be cellular cementum, covers the roots, especially in the inter-radicular area [18,28]. Dentin is normal, with well-formed dentinal tubules, but the dentino-enamel junction lacks its characteristic scalloped configuration [27,33,34].

#### Gingival phenotype

**Figure 4:**
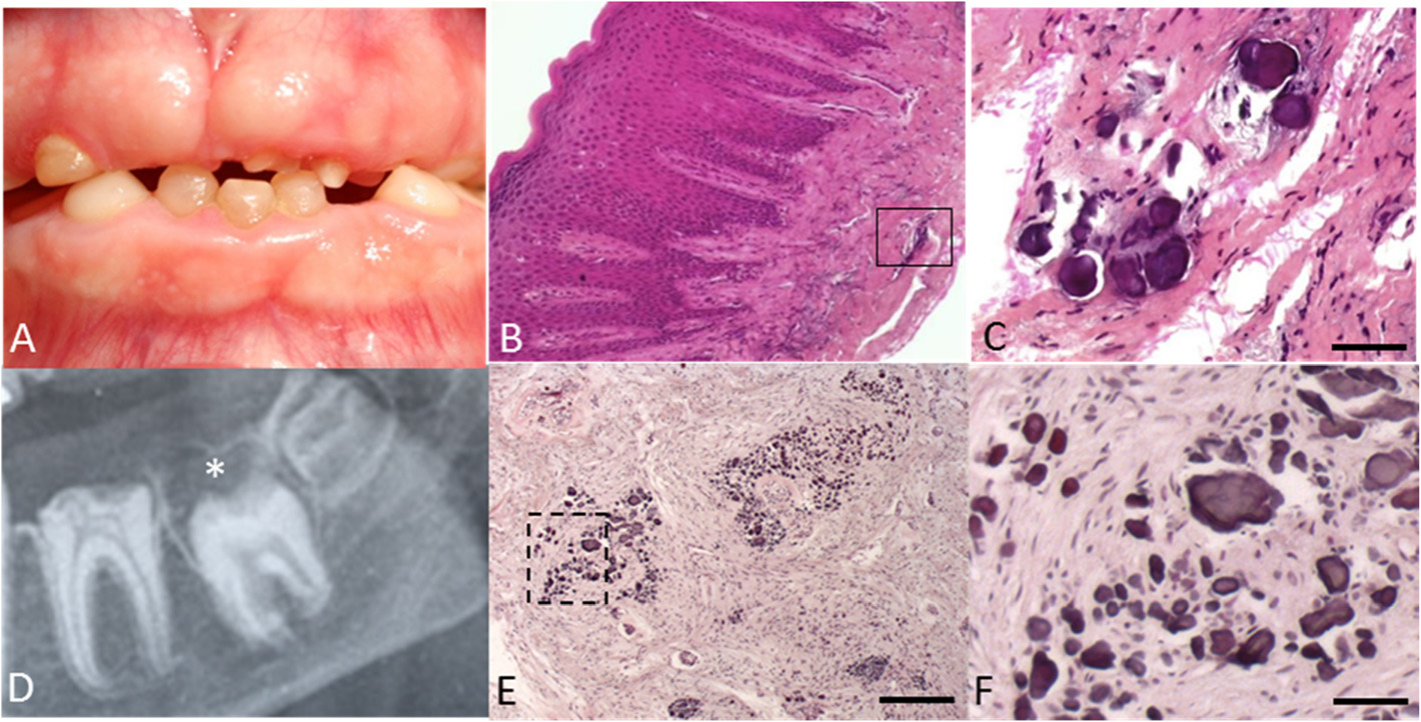
**Gingival and dental-follicle ectopic calcifications in patients with ERS. (A)** Gingival hyperplasia. **(B-C)** H&E staining of resected gingiva showing ectopic calcification in connective tissue. Note the lamellar concentric layer surrounding the central core of the calcification. **(D)** Hyperplastic dental follicle well delimited by a sclerotic margin. **(E)** H&E staining of resected follicle showing a similar group of ectopic calcification. **(F)** Magnification of boxed area from E. Scale bar: **(E)**: 200 μm; **(C, F)**: 50 μm.

Fibrous gingival hyperplasia is pathognomonic, with variable severity. This explains why, when prominent, patients have been diagnosed clinically as having AIGFS. Histological analysis of gingival tissue demonstrated focal ectopic calcification [13,30]. The gingival epithelial layer is well structured with elongated and thin papillae. Gingival connective tissue appears increasingly fibrous with depth, with the occurrence of focal, round ectopic calcification near the alveolar bone (Figure 4C). Ectopic mineralization is most often observed in close proximity to odontogenic epithelium cells [35] and vascular vessels in rich collagen-containing connective tissue running in all direction [30]. Different sizes of mineralized foci range from discrete spots to confluent nodules of up to 100 μm (Figure 4C-F). Nodules present with concentric laminations with increased porosity in the center, and were suggested to be of cementoid origin [30].

Histopathological analysis of the pericoronal follicles of impacted teeth revealed calcified nodules similar to gingival ones (Figure 4E-F) [12,13,21]. Based on these features, some patients were reported to have AI associated with pericoronal hamartoma exclusively [31,33].

### Renal phenotype

Whilst the oral phenotype is evident in childhood, the renal involvement is clinically silent at this age and requires further investigation for detection (Table 1). Nephrocalcinosis was reported in patients affected by “AI and nephrocalcinosis” and by “ERS” but not explored in AIGFS patients. Renal involvement is characterised by bilateral medullary nephrocalcinosis on renal plain radiograph, ultrasound or CT imaging [8,9,12,14,16]. Renal cortex biopsies undertaken in selected patients revealed focal clusters of sclerosed glomeruli, marked periglomerular fibrosis with lymphocytic and plasma cell infiltration of the renal interstitium [9,14].

Hypocalciuria and reduced citrate excretion are typical with hypophosphatemia present less frequently [7,10,12,16]. Urine from affected patients promoted calcium oxalate (CaOx) crystal growth compared to controls [21]. Values for serum urea, creatinine and serum electrolytes, as well as creatinine clearance, alkaline phosphatase, parathyroid hormone, vitamin D are typically within normal limits.

## Aetiopathogenesis

*FAM20A* mutations cause both AIGFS and ERS [17-23]. *Fam20a*−/− mice present with the dental phenotype observed in patients with *FAM20A* mutations, i.e., enamel defects, microdontia and flat molars. The common phenotype observed in mouse and man suggests that FAM20A plays a role in enamel secretion and maturation stages, although its distinct roles in amelogenesis and nephrocalcinosis remain to be discovered. The FAM20 family includes FAM20A, FAM20B and FAM20C proteins. FAM20C is phylogenetically closer to FAM20A than FAM20B and has been more extensively studied.

FAM20C is a Golgi casein kinase that phosphorylates several secreted proteins implicated in biomineralization, including the SIBLING proteins (small integrin-binding ligand, N-linked glycoproteins) [41]. It is expressed by osteoblasts, ameloblasts (during secretion stage) and odontoblasts and plays an essential role in tooth development [18,20,21]. Mutations of *FAM20C* cause autosomal-recessive neonatal osteosclerotic bone dysplasia (Raine syndrome; OMIM 259775). FAM20B is less well understood and is not currently associated with human disease. *Fam20b* deletion in a mouse model is embryonically lethal. Embryos at E12.5 show severely stunted growth, with multisystem organ hypoplasia and delayed development, most evident in the skeletal system, eyes, lung, gastro-intestinal tract and liver [42]. Current understanding is that FAM20B is functionally important in cartilage matrix formation and skeletal development by controlling proteoglycan synthesis [43].

Based on the high sequence homology of FAM20 family members, it may be speculated that FAM20A is an additional kinase with specific targets implicated in mineralization and/or calcium transport and proteoglycan synthesis. While the reported clinical features of ERS/AIGFS primarily involve the orodental tissues and kidneys, *FAM20A* expression has been detected in additional tissues by RT-PCR: liver, lung, heart, stomach, placenta, parathyroid, thymus and kidney [44]. *Fam20a,b,c* transcripts have been detected during odontogenesis at mouse E14.5 molar and incisor cap stages [45]. *In situ* hybridization and immunolocalization performed in mouse mandibular incisors revealed *Fam20a* expression in all enamel organ cells (ameloblasts, stratum intermedium and stellate reticulum), odontoblasts, dental pulp cells and suprabasal gingival epithelium, with lower expression in the ameloblasts [20,23].

Histological analysis of *Fam20a*−/− mouse incisors demonstrated that the ameloblast layer becomes progressively disorganized. All other examined tissues (bone, dentine, cementum) appeared normal. Interestingly, in line with the hamartoma phenotype reported in some human cases [33,35], the enamel organ appeared disorganized, forming odontogenic tumor-like structures [42]. A similar phenotype was described in *Msx2*−/− mice, raising the possibility that MSX2 and FAM20A function within a shared molecular pathway [46]. Ectopic calcification was prominent in the kidneys of *Fam20a*−/− mice, but was also present in muscular arteries, lungs, heart, eyes, pancreas, thalamus, uterus, choroid plexus, skeletal muscle, and cutaneous tissue. Blood levels of calcium and phosphate were normal [42].

## Diagnostic criteria of ERS/AIGFS

Clinical diagnosis focuses on the association of orodental features and renal findings that may lead on to renal impairment. Ectopic lung mineralization has been reported in one patient [21]. However, the oral phenotype is characteristic and, in the absence of other co-segregating health problems, sufficient to clinically diagnose ERS (Figure 1) and direct genetic testing to look for *FAM20A* recessive mutations (Table 1).

Orodental clinical features typical of *FAM20A* recessive mutations include:

1) generalized thin hypoplastic or absent enamel;

2) primary and permanent teeth affected;

3) flat cusps on posterior teeth;

4) relative microdontia and spaced teeth;

5) intra-pulpal calcifications;

6) delayed tooth eruption;

7) impacted posterior teeth with hyperplastic follicle (hamartoma-like) and altered eruption pathway;

8) root dilacerations of impacted teeth;

9) gingival fibromatosis (variable severity);

10) gingival and dental follicle ectopic calcification on biopsies;

Additional features that may be observed include:

11) semi-lunar shape of central incisor edge;

12) crown resorption of non-erupted teeth;

13) anterior open-bite;

14) root hypercementosis and inter-radicular dentine dysplasia,

15) supernumerary teeth.While individual features are not specific to ERS, they are pathognomonic when they occur together in children in the absence of other developmental abnormalities. Nephrocalcinosis is not always detected, in part reflecting that it is not investigated rather than due to its absence. We cannot exclude the possibility that it is not detectable in children. Using the orodental clinical features listed above, diagnosis can be made as early as 5 years of age, based solely on enamel defects and radiographic features observed on the panoramic radiograph. The dental radiographic images are per se diagnostic (Figure 1I). Subsequently, such patients should be referred to a nephrologist for evaluation and follow-up as well as to a clinical geneticist.

## Differential diagnosis

AI occurs either in isolation or as part of a syndrome (such as Jalili syndrome [47], Raine syndrome [48], epidermolysis bullosa [49], tricho-dento-osseous syndrome [50]). A review of literature published since 1972 shows several reports with confusing and incomplete characterization of ERS. Severe hypoplastic enamel constitutes the first element of differential diagnosis. Hypomaturation or hypocalcified AI have never been described in ERS. Multiple diagnostic features, as described above, should be present together. Isolated association, such as AI and gingival fibromatosis, AI and hamartoma, and AI and tooth delayed eruption, may reflect other rare AI diseases. Nibali *et al.*, for example, described patients with gingival hyperplasia and AI [51]. Enamel appeared as in hypomaturation type AI with diffuse opacities covering the entire crown. No root or crown dysmorphology was described and pulp stones were absent. Eruption pathways and timing were normal, arguing for a diagnosis distinct from ERS. AI and hamartoma has been reported isolated or in several syndromes such as Cowden syndrome [52], Von Recklinghausen disease [53], and familial tuberous sclerosis [54]. Only a few cases of them appear to correspond to a complete description of ERS (Table 1 and Additional file 1) [31,32,35].

Finally, renal disease and enamel defects can also be present in pathologies other than ERS. Enamel defects are a frequent finding (58.3%) in patients affected by renal disorders [55]. Indeed, the kidney is central to regulation of calcium and phosphate homeostasis. Different renal proteins previously known to play a role in the systemic pH homeostasis in mammals, have also been described to be expressed during amelogenesis: the carbonic anhydrase II [56], the acid–base exchangers AE2, NBCe1 and NHE1 [57,58]. Suda *et al.* described a patient with severe AI and polycystic kidney disease leading to nephrocalcinosis [15], caused by a mutation in *MSX2*. Panoramic radiography revealed no pulpal stones and no eruption anomalies. Fu *et al.* described a patient with ERS associated with hypokalemic metabolic alkalosis and impaired renal concentration [39]. Besides nephrocalcinosis, the dental and renal phenotypes did not correspond with those seen in ERS, suggesting this patient was wrongly diagnosed with ERS. This reinforces the importance of a detailed oral clinical examination, rather than exclusively focusing on AI.

## Genetics and genetic counseling

Combining homozygosity mapping and whole exome sequencing, O’Sullivan *et al.* identified the first homozygous mutation in *FAM20A* in a consanguineous family with AIGFS [20]. To date, 40 (33 unique) recessive *FAM20A* mutations have been identified in 29 ERS families (MIM#204690) (Table 2), ERS/AIGFS is a recessive inherited disease with either homozygous or combined heterozygous *FAM20A* mutations and is likely one unique syndrome. Heterozygous carriers appear to be phenotypically healthy. *FAM20A* is located on chromosome 17q24.2, is 65,839 base pairs long and consists of 11 exons. All but 2 of the 33 identified mutations lead to premature stop codons (Table 2). The remaining two are non-synonymous mutations in well-conserved regions of the protein [18].

**Table 2:** **List of all *FAM20A *** **mutations reported in the literature**

**Location**	**cDNA**	**Protein**	**Mutation status**	**Reference**	**Patient**	**Number of patients**	**Sex**
Exon 1	c.34_35delCT	p.Leu12Alafs*67	Hom	Cho *et al.*[19]	Family 1	1	1 Female
Exon 1	c.34_35delCT	p.Leu12Alafs*67	Hom	Jaureguiberry *et al.*[17]	Family 6	1	1 Male
Exon 1	c.34_35delCT	p.Leu12Alafs*67	Het	Jaureguiberry *et al.*[17]	Family 16	2	2 Females
Exon 1	c.174-175ins29	p.Arg59Argfs*85	Hom	Cabral *et al.*[22]	Family 1	12	10 Males; 2 Females
Exon 1	c.217C > T	p.Arg73*	Het	Jaureguiberry *et al.*[17]	Family 11	2	1 Male; 1 Female
Exon 1	c.349_367del19	p.Leu117Cysfs*22	Het	Kantaputra *et al.*[21]	Family 1	1	1 Male
Intron 1	c.405-1G > C		Het	Wang *et al.*[23]	Family 1	1	1 Female
Exon 2	c.406C > T	p.Arg136*	Hom	O’Sullivan *et al.*[20]	Family 1	4	1 Female
Exon 2	c.406C > T	p.Arg136*	Het	Wang *et al.*[18]	Family 3	2	2 Males
Exon 2	c.406C > T	p.Arg136*	Hom	Jaureguiberry *et al.*[17]	Family 5	1	1 Female
Exon 2	c.518 T > G	p.Leu173Arg	Hom	Jaureguiberry *et al.*[17]	Family 9	1	1 Male
Intron 2	c.589 + 1G > A		Het	Jaureguiberry *et al.*[17]	Family 2	2	1 Male; 1 Female
Intron 2	c.590-2A > G	p.Asp197_Ile214delinsVal	Het	Cho *et al.*[19]	Family 4	1	1 Female
Exon 3	c.612delC	p.Leu205Cysfs*11	Het	Jaureguiberry *et al.*[17]	Family 16	2	1 Female
Exon 4	c.641_719del	p.Ile214Asnfs*46	Het	Jaureguiberry *et al.*[17]	Family 13	2	1 Male; 1 Female
Intron 4	c.719 + 1G > C		Het	Jaureguiberry *et al.*[17]	Family 3	2	1 Male; 1 Female
Intron 4	c.720-2A > G	p.Gln241_Arg271del	Hom	Wang *et al.*[18]	Family 2	1	1 Male
Exon 5	c.727C > T	p.Arg243*	Het	Jaureguiberry *et al.*[17]	Family 10	1	2 Female
Exon 5	c.727C > T	p.Arg243*	Het	Jaureguiberry *et al.*[17]	Family 11	2	1 Male; 1 Female
Exon 5	c.755_757delTCT	p.Phe252del	Het	Jaureguiberry *et al.*[17]	Family 13	2	1 Male; 1 Female
Intron 5	c.812 + 2 T > G		Hom	Jaureguiberry *et al.*[17]	Family 14	1	1 Female
Intron 5	c.813-2A > G	p.Arg271Serfx*70	Hom	Cho *et al.*[19]	Family 2	4	3 Males; 1 Female
Exon 6	c.826C > T	p. Arg276*	Het	Cho *et al.*[19]	Family 4	1	1 Female
Exon 6	c.907_908delAG	p.Ser303Cysfs*76	Hom	Jaureguiberry *et al.*[17]	Family 15	3	2 Males; 1 Female
Exon 6	c.913_914delTT	p.Phe305Leufs*74	Het	Jaureguiberry *et al.*[17]	Family 2	2	1 Male; 1 Female
Exon 6	c.915_918delCTTT	p.Phe305Leufs*76	Hom	Jaureguiberry *et al.*[17]	Family 1	1	1 Male
Exon 6	c.915_918delCTTT	p.Phe305Leufs*76	Het	Kantaputra *et al.*[21]	Family 1	1	1 Male
Exon 7	c.992G > A	p.Gly331Asp	Hom	Wang *et al.*[18]	Family 1	3	1 Male 2 Females
Exon 8	c.1175_1179delGGCTC	p.Arg392Profs*22	Hom	Cho *et al.*[19]	Family 3	2	2 Males
Exon 8	c.1207G > A	p.Asp403Asn	Het	Wang *et al.*[23]	Family 1	1	1 Female
Exon 9	c.1228_1229delGA	p.Asp410Profs*5	Het	Jaureguiberry *et al.*[17]	Family 10	1	1 Female
Intron 9	c.1302-2A > G		Het	Kantaputra *et al.*[21]	Family 2	1	1 Female
Exon10	c.1348_1349delTC	p.Ser450Profs*20	Het	Jaureguiberry *et al.*[17]	Family 3	2	1 Male; 1 Female
Intron 10	c.1361 + 4del		Hom	Wang *et al.*[23]	Family 2	2	1 Male; 1 Female
Exon 11	c.1369A > T	p.Lys457*	Hom	Jaureguiberry *et al.*[17]	Family 12	1	1 Female
Exon 11	c.1432C > T	p.Arg478*	Hom	Jaureguiberry *et al.*[17]	Family 8	1	1 Male
Exon 11	c.1432C > T	p.Arg478*	Het	Wang *et al.*[18]	Family 3	2	1 Male; 1 Female
Exon 11	c.1480delC	p.His494fs*13	Het	Kantaputra *et al.*[21]	Family 2	1	1 Female
Exon 11	c.1475_1482dupAACCCCAC	p.Leu495Asnfs*15	Hom	Jaureguiberry *et al.*[17]	Family 4	2	1 Male; 1 Female
Exon 11	c.1513delA	p.Ile505Serfs*2	Hom	Jaureguiberry *et al.*[17]	Family 7	2	1 Male; 1 Female
Total reported	40 (33 unique mutations)		18 Hom; 22 Het		29 Families	60 index cases	32 Males; 28 Females

Hom: homozygous; Het: heterozygous; *premature stop codon.

## Natural history and prognosis

Pediatric dentists are usually the first health practitioners to see affected patients due to AI and delayed eruption of permanent teeth. Due to the association of this specific oral phenotype with nephrocalcinosis, patients should be referred to a nephrologist for assessment. The natural evolution of nephrocalcinosis associated with *FAM20A* mutations is not well established. Two patients were reported to have died prematurely before the age of thirty: one had coronary occlusion and the other developed chronic pyelonephritis [5]. One author reported that the medullary calcification became coarser and denser as his patients aged from 8 years to 14 years old [10]. Three patients experienced acute or chronic pyelonephritis, 1 had urinary calculus [7,9,14] and 1 developed chronic renal failure [8]. For the remaining patients with nephrocalcinosis, no anomalies or major complications were reported [11-13,17], in line with the clinical history of *Fam20a*−/− mutant mice, which depicted a partial resolution of ectopic mineralization in muscular arteries and lungs as the mice matured [42]. A regular follow-up of the nephrocalcinosis and evaluation of kidney function throughout childhood and adulthood may enable the initiation of preventive treatment before the occurrence of renal failure.

Except for gingival hyperplasia, for which gingivectomy is successful with no recurrence reported, the orodental phenotype is often complex to manage [30,33]. AI results in considerable morbidity; affected individuals have teeth with poor function and aesthetics, have lower self-esteem and report an inferior quality of life [59]. Dental wear and aesthetics are the main motives for the initial consultation, but absence of eruption is the most challenging problem to solve. Only erupted teeth can be restored with conventional treatments such as ceramic crowns for example [8]. More severe cases are however characterized by a large number of non-erupted teeth, forcing complete rehabilitation with overdentures. Long-term follow-up reveals that teeth with follicle hyperplasia do not erupt and the extraction of primary teeth does not facilitate the eruption of permanent teeth. Orthodontic traction has been tested to bring them in occlusion, but results have been slow and inconsistent. The absence of a global therapeutic consensus concerning non-erupted teeth complicates dental surgery.

## Conclusion

A careful review of the published literature and case reports highlights a significant overlap in the oral phenotype between cases of AI with hamartomas, unerupted teeth, AIGFS and ERS. Recently, recessive mutations in *FAM20A* were shown to be responsible for both AIGFS and ERS. Supported by the phenotype observed in *Fam20a* null mice, human phenotypic and genetic data suggest that, rather than being allelic, ERS and AIGFS are in fact the same syndrome. The phenotype is characterized by severe enamel hypoplasia, delayed or absent tooth eruption, ectopic eruption pathway, and pulp and gingival calcification in temporary and permanent dentition. Gingival hyperplasia typically accompanies these features but is more variable, ranging from discrete to severe. We propose that all affected patients be categorized under the term ERS, and that the 2 OMIM entries (ERS: MIM#204690, AIGFS: MIM#614253) be fused. Since the oral phenotype in the absence of other developmental health problems is pathognomonic, dentists have a key role in the diagnosis and referral of patients to both nephrologists for renal assessment and to geneticists to identify the causative *FAM20A* mutations.

## Abbreviations

AI: Amelogenesis Imperfecta; AIGFS: Amelogenesis imperfecta and gingival fibromatosis syndrome; ERS: Enamel renal syndrome; FAM20A: Family with sequence similarities 20 member A; OMIM: Online mendelian inheritance in man.

## Competing interests

The authors declare that they have no competing interests.

## Authors’ contributions

MDM, MQ, ACA, wrote the manuscript. AM, MV, AB, ABZ, MU contributed towards the revision of the manuscript. All authors read and approved the final manuscript. MU and MQ explored the genotype of AI patients.

## Supplementary Material

Additional file 1 Detailed description of oral phenotype of reported cases with clinical features potentially describing Enamel Renal Syndrome. Click here for file
