# Supplementary material for: Pathognomonic oral profile of Enamel Renal Syndrome (ERS) caused by recessive FAM20A mutations
Source: Orphanet J Rare Dis. 2014 Jun 14;9:84. doi: 10.1186/1750-1172-9-84 (PMC4071802; doi:10.1186/1750-1172-9-84)
Supplement: Additional file 1 — Detailed description of oral phenotype of reported cases with clinical features potentially describing Enamel Renal Syndrome. [file 1750-1172-9-84-S1.pdf]

	Number of reported		Age of diagnosis	Oral phenotype														Kidney phenotype	Diagnosis reported
	Families	Cases		Enamel hypoplasia / aplasia	Delayed eruption / unerupted teeth	Intra-pulpal calcifications	Crown resorption of unerupted teeth	Semi-lunar shaped defect of central incisor	Inter-radicular dentinal dysplasia	oot hypercementos	Root dilaceration	Tooth follicle hyperplasia	Tooth follicle calcification	Gingival hyperplasia	Gingival calcification	Anterior open-bite	Eruption corridor modified	Bilateral nephrocalcinosis	
MacGibbon, 1972	1	1	25y	+	+	+	+	+		+	+							+	Generalized **enamel hypoplasia** and **renal** dysfunction.
Chosack *et al.* , 1979	1	4	5y, 13y, 15y, 18y	+	+	+	+				+	+						Not investigated	**Amelogenesis imperfecta** among Israeli Jews and the description of a new type of local **hypoplastic autosomal recessive amelogenesis imperfecta.**
Lubinsky *et al.* , 1985	1	2	10y, 12y	+	+	+	+	+		+	+	+						+	Syndrome of **amelogenesis imperfecta**, **nephrocalcinosis**, impaired renal concentration, and possible abnormality of calcium metabolism.
Nakata *et al.* , 1985	1	2	13y,12y	+	+		+	+	+	+ (1/2)	+	+	+	+	+			Not investigated	**Interradicular dentin dysplasia** associated with **amelogenesis imperfecta.**
Ooya *et al.* , 1988	1	1	12y	+	+	+	+	+						+	+			Not investigated	Autosomal recessive rough hypoplastic **amelogenesis imperfecta**. A case report with clinical, light microscopic, radiographic, and electron microscopic observations.
Van Heerden *et al.* , 1990	2	2	14y; 26y	+	+	+	–		+	+	+	+	+					Not investigated	**Amelogenesis imperfecta**: multiple impactions associated with **odontogenic fibromas (WHO) type**.
Peters *et al.,* 1992	1	1	26y	+	+	+	+			+	+	+	+	+				Not investigated	Rough hypoplastic **amelogenesis imperfecta** with **follicular hyperplasia**.
Hall *et al.* , 1995	1	2	10y6m, 14y8m	+	+	+	+	+			+	–		+		+ (1/2)		+	**Amelogenesis imperfecta** and **nephrocalcinosis** syndrome. Case studies of clinical features and ultrastructure of tooth enamel in two siblings.
Phakey *et al.* , 1995																			Ultrastructural study of tooth enamel with **amelogenesis imperfecta** in AI-**nephrocalcinosis** syndrome.
Dellow *et al.* , 1998	1	2	8y/32y, 12/40y (dental/kidney)	+	+	+	+											+	**Amelogenesis imperfecta**, **nephrocalcinosis**, and hypocalciuria syndrome in two siblings from a large family with consanguineous parents.
Raubenheimer and Noffke, 2002	1	1	19y	Enamel dysplasia	–	–	–					COFs (no tooth associated)	COFs calcifications			+		Not investigated	**Enamel dysplasia, hamartomas.**
Normand de La Tranchade *et al.* , 2003	1	1	15y	+	+	+	+	–				+	+	+				+	**Amelogenesis imperfecta** and **nephrocalcinosis**: a new case of this rare syndrome.
Paula *et al.* , 2005	1	1	13y	+	+	+	–	+			+	+	+	+				+	Case report of a rare syndrome associating **amelogenesis imperfecta** and **nephrocalcinosis** in a consanguineous family.
Cetrullo *et al.* , 2006	1	2	18y,14y	+	–	–	–	–			–	–		–		+		+	Two cases of familial hypomagnesemia with hypercalciuria and **nephrocalcinosis**: dental findings.
Feller *et al* ., 2006	1	1	12y	+	+	+		–		+		+	+	+	+	+		Not investigated	**Enamel dysplasia** with **odontogenic fibroma-like hamartomas**: review of the literature and report of a case.
Fu *et al.* , 2006	1	1	14y	+	–		–							–				+	**Enamel-renal syndrome** associated with hypokalaemic metabolic alkalosis and impaired renal concentration: a novel syndrome?
Elisabeth *et al.,* 2007	2	2	23y; 20y	+	+	+	+				+	+		–				+	**Amelogenesis imperfecta** with **renal disease**--a report of two cases.
Feller *et al.* , 2008	1	1	20y	+	+	+				+		+	+	+	+	+	+	Not investigated	**Enamel dysplasia hamartomatous atypical follicular hyperplasia**: review of the literature and report of a case.
Roquebert *et al.* , 2008	1	1	10y	+	+	+	+		+	+	+	+	+	+	+		+	Not investigated	**Amelogenesis imperfecta**, **rough hypoplastic type**, **dental follicular hamartomas and gingival hyperplasia**: report of a case from Central America and review of the literature.
Martelli-junior *et al.* , 2008	1	4	19,13y,13y,18y	+	+	+	+	+			+ (1/4)	+	+	+	+			No history of renal pathology (but not investigated)	Case reports of a new syndrome associating **gingival fibromatosis** and **dental abnormalities** in a consanguineous family.
Martelli-Junior *et al.* , 2011	1	1	9y	+	+	+		+				+		+				+	**Amelogenesis imperfecta** and **nephrocalcinosis** syndrome: a case report and review of the literature.
Dos Santos *et al.* , 2011	1 [1]	4	20y,15y,15y,20y	+	+	+	+				+	+		+	+		+	Not investigated	Imaging evalution of the **gingival fibromatosis** and **dental abnormalities** syndrome.
Cho *et al.,* 2011	4	4		+	+	+		+			+	+		+				Not investigated	Novel **FAM20A** mutations in hypoplastic **amelogenesis imperfecta**.
Kala Vani *et al.* , 2012	1	1	11y	+	+		–					+		–		+		+	**Enamel renal syndrome**: a rare case report.
Hegde *et al* ., 2012	1	2	20y, 17y	+	+	+	+	+	+		+	+		+			+	Not investigated	**Multiple Unerupted Teeth** with Amelogenesis Imperfecta in Siblings
O'Sullivan *et al.* , 2012	1 [1]	4	19y,13y,13y,18y	+	+	+	+	+			+ (1/4)	+	+	+	+			Not investigated	Whole-Exome sequencing identifies **FAM20A** mutations as a cause of **amelogenesis imperfecta** and **gingival hyperplasia** syndrome.
Jaureguiberry *et al.* , 2012	16 [2]	25	from 12y to 64y	+	+	+	+	+			+	+	+	+	+		+	+	**Nephrocalcinosis** (**Enamel Renal Syndrome**) caused by autosomal recessive **FAM20A**
Wang *et al.* , 2013	3	5		+	+	+	+	+	+	+	+	+		+		+	+	+	**FAM20A** Mutations Can Cause **Enamel-Renal Syndrome (ERS)**
Cabral *et al.* , 2013	1	12	N.A.	+	+									+				+	Autosomal recessive **gingival hyperplasia** and **dental anomalies** caused by a 29-base pair duplication in the **FAM20A** gene
Wang *et al. (b)* , 2013	2	3	12y; 10y	+	+	+	+	+			+	+		+		+	+	+ (1/2) Neg. US in proband of Family 1	**FAM20A** Mutations Associated with **Enamel Renal Syndrome**
Kantaputra et al., 2013	2	2	14y; 10y	+	+	+	+	+				+	+	+	+		+	+	**Enamel-Renal-Gingival Syndrome** and **FAM20A** Mutations

COF: Central Odontogenic Fibroma
Neg. US: Negative ultrasounds analysis
[1]: Family previously described in Martelli-Junior et al., 2008
[2] : Two families were previously described (Dellow et al, 1998; Paula et al., 2005)
[3]: Family sequenced in Jaureguiberry *et al* ., 2012
[4]: Family sequenced in O'Sullivan et al., 2012

FAM20A mutation
expected
expected for family 2 only
expected
expected
expected
expected
expected
expected
confirmed[3]
not expected
expected
confirmed[3]
not expected
expected
not expected
expected for case 1 only
expected
expected
expected
expected
confirmed[4]
confirmed
expected
expected
confirmed
confirmed
confirmed
confirmed
confirmed
confirmed
